# Health Care Worker Usage of Large-Scale Health Information Exchanges in Japan: User-Level Audit Log Analysis Study

**DOI:** 10.2196/56263

**Published:** 2024-10-09

**Authors:** Jun Suzumoto, Yukiko Mori, Tomohiro Kuroda

**Affiliations:** 1 Graduate School of Medicine, Kyoto University, Kyoto, Japan; 2 Division of Medical Information Technology and Administration Planning, Kyoto University Hospital, Kyoto, Japan; 3 Graduate School of Informatics, Kyoto University, Kyoto, Japan

**Keywords:** health information exchange, audit log, Japan, HIE, audit, logs, usage, medical informatics, rate, hospitals, electronic health record

## Abstract

**Background:**

Over 200 health information exchanges (HIEs) are currently operational in Japan. The most common feature of HIEs is remote on-demand viewing or searching of aggregated patient health data from multiple institutions. However, the usage of this feature by individual users and institutions remains unknown.

**Objective:**

This study aims to understand usage of the on-demand patient data viewing feature of large-scale HIEs by individual health care workers and institutions in Japan.

**Methods:**

We conducted audit log analyses of large-scale HIEs. The research subjects were HIEs connected to over 100 institutions and with over 10,000 patients. Each health care worker’s profile and audit log data for HIEs were collected. We conducted four types of analyses on the extracted audit log. First, we calculated the ratio of the number of days of active HIE use for each hospital-affiliated doctor account. Second, we calculated cumulative monthly usage days of HIEs by each institution in financial year (FY) 2021/22. Third, we calculated each facility type’s monthly active institution ratio in FY2021/22. Fourth, we compared the monthly active institution ratio by medical institution for each HIE and the proportion of cumulative usage days by user type for each HIE.

**Results:**

We identified 24 HIEs as candidates for data collection and we analyzed data from 7 HIEs. Among hospital doctors, 93.5% (7326/7833) had never used HIEs during the available period in FY2021/22, while 19 doctors used them at least 30% of days. The median (IQR) monthly active institution ratios were 0.482 (0.470‐0.487) for hospitals, 0.243 (0.230‐0.247) for medical clinics, and 0.030 (0.024‐0.048) for dental clinics. In 51.9% (1781/3434) of hospitals, the cumulative monthly usage days of HIEs was 0, while in 26.8% (921/3434) of hospitals, it was between 1 and 10, and in 3% (103/3434) of hospitals, it was 100 or more. The median (IQR) monthly active institution ratio in medical institutions was 0.511 (0.487‐0.529) for the most used HIE and 0.109 (0.0927‐0.117) for the least used. The proportion of cumulative usage days of HIE by user type was complex for each HIE, and no consistent trends could be discerned.

**Conclusions:**

In the large-scale HIEs surveyed in this study, the overall usage of the on-demand patient data viewing feature was low, consistent with past official reports. User-level analyses of audit logs revealed large disparities in the number of days of HIE use among health care workers and institutions. There were also large disparities in HIE use by facility type or HIE; the percentage of cumulative HIE usage days by user type also differed by HIE. This study indicates the need for further research into why there are large disparities in demand for HIEs in Japan as well as the need to design comprehensive audit logs that can be matched with other official datasets.

## Introduction

A health information exchange (HIE) is an electronic mobilization system of clinical data across entities such as institutions or organizations, or an organization that controls such systems [1-3]. The appropriate sharing of medical information using HIEs enables fewer duplicated procedures, less duplicated imaging, and fewer total orders. HIE usage is also associated with improved medication reconciliation and immunization [1,4,5]. HIEs have several major features. The first feature enables sharing (ie, sending and receiving) of secure information electronically between care providers to support coordinated care, known as directed exchange [1,6]. The second enables remote, on-demand viewing or searching of aggregated patient health data from multiple health care institutions. This feature is known as query-based exchange or query-based HIE [1,6,7]. In addition to these two features, consumer-mediated exchange allows patients to aggregate and control the use of their health data among providers [1].

In Japan, HIEs are not widely used. Instead, systems called “Chiiki iryo joho renkei nettowa-ku” have provided features equivalent to HIEs [8-11]. In some literature, these are called “regional health care networks” (RHNs) in English [10]. Past surveys show that over 200 RHNs of various sizes operate in Japan [9]. Most of these RHNs are sponsored by governments or local authorities. According to a Ministry of Health, Labour and Welfare (MHLW) report, 27 RHNs cover entire prefectures, 104 RHNs are within the secondary medical area, 32 are the size of a municipality, and 15 are smaller than a municipality [9]. Item 2.10.2 of the Japan Medical Association Research Institute’s 2021 survey [8] investigates the services provided by 229 RHNs, revealing that “sharing of medical data” was the most common service, provided by 190 (83%) RHNs, followed by “sharing of medical images,” provided by 187 (81.6%). These features are equivalent to a query-based exchange. In addition, 66 (28.8%) RHNs provide email services and 45 (19.7%) provide electronic patient referral documents, which is equivalent to a directed exchange. Only 9 (3.9%) RHNs provide self-management systems for patients, which is equivalent to a consumer-mediated exchange. In other words, query-based exchange is the most common feature of Japanese HIEs. Since HIEs and RHNs essentially refer to systems with the same features, we will refer to RHNs as HIEs in the subsequent paragraphs. To avoid confusion between computer systems and organizations, we refer to the organization that promotes HIEs as a regional health information organization (RHIO), a term adopted in most literature [7,12].

It is crucial to evaluate the benefits of HIEs for individual health care workers and institutions. Review papers on HIEs have highlighted the importance of understanding whether the system is used [13]. To study the actual use of HIEs, audit logs have often been analyzed [14-25]. The Japanese Association of Healthcare Information Systems Industry published a technical document called “JAHIS’s Guide Ver.1.0 on Evaluation Indicators for Regional Medical Collaboration” [26], which emphasizes the importance of evaluating HIE systems using audit log analysis. However, most previous reports [8,9,27] or peer-reviewed journal articles [28-30] about HIE in Japan either did not include audit log analysis or were limited to simple analyses. The MHLW conducted a survey [9] on access to HIEs in 2019. The average monthly active institution ratio based on the MHLW report was 0.381 (SD 0.199) for HIEs connected to 100 or more institutions (Table S1 in Multimedia Appendix 1), meaning that more than half of the connected institutions did not access HIEs. Although the MHLW report suggested low utilization of large-scale HIEs, it did not include a user-level analysis. As there are no studies analyzing the audit logs of multiple HIEs in Japan at the user level, the usage of HIE by individual users or medical institutions remains unknown.

The primary objective of this study was to clarify the extent to which query-based exchange is used by individual health care workers and institutions in large-scale HIEs in Japan by analyzing audit logs at the user level. One reason for investigating only query-based exchange is that, as already mentioned, it is the most common feature of such systems in Japan. The other reason is that, while directed exchange has alternatives such as patient referral letters on paper and consumer-mediated exchange has alternatives such as prescription records on paper, query-based exchange can only be achieved through HIEs. Therefore, analyzing the usage of this feature indicates the significance of HIEs. There are two reasons for investigating only large-scale HIEs. First, it is not realistic to investigate the audit logs of all 200 or more HIEs. Second, large-scale HIEs appear to have spread, as they are accepted by many medical institutions and many patients in the region. When conceptualizing this study, we thought that by investigating large-scale HIEs, we would be able to make suggestions for the increased use of small-scale HIEs.

## Methods

### Study Design and Data Collection

In this study, we collected data on HIEs that met the following inclusion criteria: (1) they must be included in the list of the survey report, “About the current situation of regional healthcare network” [9] published by the MHLW, and (2) each HIE must be connected to more than 100 institutions and have more than 10,000 patients according to the report above. We asked all RHIOs operating HIEs that met the inclusion criteria to cooperate in this study. When requesting data from each RHIO, we promised to conceal the identity of the RHIO that provided the data in this study. We also agreed to present our published analysis and results in such a way that the RHIO that provided the data would be concealed. This was done to avoid any potential effects that public disclosure of the usage status of each HIE would have on its operation. We obtained data from RHIOs that provided informed consent.

The profile and audit log data of health care workers enrolled in the HIE were collected. In this study, we did not collect patient data from the HIE. Textbox 1 displays the data requested for each HIE; we only received the available data each RHIO could provide. Consequently, datasets and data representation formats differ among HIEs. We also obtained data on the number of connected institutions per month or year for each RHIO. The maximum period of the audit log data was 5 years. We aimed to acquire audit log data from April 1, 2017, to March 31, 2022, but if there were no accumulated data for that period, we asked the RHIO to provide data for the period that could be extracted.

Textbox 1. **User data analyzed in this study.**

1. Occupation
2. Institution
3. Anonymous identifier
4. Date of account registration and account deletion in health information exchange
5. Date and time of access to query-based exchange
6. Type of data accessed by the user
7. Type of device used for access

### Ethical Considerations

This study was approved by the ethics committee of Kyoto University Graduate School and the Faculty of Medicine. The accession number was R3266-7. The disclosure document regarding the research plan and the data to be extracted were published on the Kyoto University Hospital website [31], ensuring that research subjects had the opportunity to opt out. Personal data obtained in the study were pseudonymized by the HIEs that provided the data. Research subjects did not receive compensation.

### Measures and Data Analyses

#### Overview

We refer to “viewing patient medical data using query-based exchange” as “HIE use” in this study. Patient medical data viewed by health care workers can be obtained from multiple storage locations such as hospital electronic medical records (EMRs). If a patient agrees to the disclosure of their medical data stored by the institution, the institution or the RHIO office will take steps to release the stored medical data to HIEs. The types of medical data disclosed by institutions and RHIOs to HIEs vary by institution. Patients can also choose the types of facilities to which their medical data can be disclosed. Several models have been proposed by the MHLW for viewing the medical data disclosed in this way [11]. For example, doctors at a clinic can see which medical tests a patient has previously had when they visit a clinic for the first time. Patients referred from a clinic to a hospital can later check at the clinic the kind of treatment they will receive at the hospital to which they were referred. We investigated how often these types of use cases presented by the MHLW occur by analyzing audit logs. Audit logs for logging into the HIE system are not subject to analysis. Furthermore, the sending and receiving of documents between medical workers using the directed exchange was not included in the analysis.

As a unit for measuring access, 1 man-day was defined as HIE use on 1 day with 1 user account. The cumulative man-days for an institution are the cumulative number of days of HIE use by each user belonging to the institution. For example, consider use by a virtual clinic within a given month. At that clinic, one doctor used HIE on 3 days, and one nurse used HIE on 2 days, which was the clinic’s total use of HIE for that month. In this case, the clinic’s usage for that month was 5 man-days. If multiple users share a common account, this aggregation methodology may underestimate HIE usage. However, sharing accounts is generally not recommended when using HIEs.

We classified the institutions enrolled in HIEs as hospitals, medical clinics, dental clinics, pharmacies, visiting nursing stations, or nursing facilities. The institutions that could not be classified into these categories were excluded from the analysis; for example, this study did not analyze public institutions such as fire departments, public health centers, local medical associations, or vendors that developed HIEs.

In this study, the financial year (FY) is from April 1 of one year to March 31 of the next year. For example, FY2021/22 started on April 1, 2021, and ended on March 31, 2022. We used R (version 4.3.1; R Foundation for Statistical Computing) to perform the analysis.

#### Percentage of Days of HIE Use by Each Hospital Doctor in FY2021/22

For each user account of doctors affiliated with hospitals, we calculated the ratio of the number of days of active HIE use. We analyzed the data of HIEs that met the following two criteria: (1) audit log data for all periods in FY2021/22 were available, and (2) each user’s date of account registration and date of account deletion in the HIE were available (Textbox 1). For all HIEs that met the criteria, the annual number of days of HIE use by each hospital doctor’s account in FY2021/22 was counted. Next, we calculated the number of days that the doctor could use HIEs in FY2021/22. For each doctor’s account that was registered with the HIE during FY2021/22, we subtracted from 365 the number of days from April 1, 2021, to the day before the account registration date. For accounts that were removed from the HIE during FY2021/22, we subtracted the number of days from the day after the account deletion date to March 31, 2022, from the remaining number of days. The number of days remaining after these subtractions is the number of days that the doctor could use the HIE in FY2021/22. For accounts that were able to use the HIE on all days in FY2021/22, we used 365 as the number of days that the account could use the HIE. We then calculated the ratio of days of HIE use by each hospital doctor. The ratio of days of HIE use was defined as follows:


(1) $$\begin{array}{c} \frac{Doctor^{\prime }s\;annual\;number\;of\;days\;of\;HIE\;use\;in\;FY2021/22}{Number\;of\;days\;that\;the\;doctor\;could\;use\;HIE\;in\;FY2021/22} \end{array}$$

#### Man-Days for Monthly HIE Use by Each Institution in FY2021/22

We calculated the man-days for monthly HIE use by each institution for each month and aggregated them by facility type. We analyzed the data of HIEs that met the following two criteria: (1) the audit log data for all periods in FY2021/22 were available, and (2) data were available on the number of participating institutions by facility type, matching our facility classification. For each institution belonging to any HIEs that meet the criteria, man-days for monthly HIE use in FY2021/22 were aggregated. Next, by each facility type, we tallied the number of months for each man-day group divided into 5 or 10 increments. Finally, for each facility type, the percentage of each man-day group was calculated.

#### Monthly Active Institution Ratio in FY2021/22

We calculated the monthly active institution ratio for each facility type, defining it as follows:


(2) $$\begin{array}{c} \frac{Number\;of\;institutions\;at\;which\;any\;members\;used\;HIE\;during\;the\;month}{Number\;of\;institutions\;participating\;in\;HIE\;at\;the\;end\;of\;the\;month}. \end{array}$$

We analyzed the data of HIEs that met the following two criteria: (1) audit log data for all periods in FY2021/22 were available, and (2) data on the number of participating institutions by facility type, which matched our facility classification, were available. For each facility type, the total number of participating institutions for the last day of each month in FY2021/22 was aggregated in all HIEs that met the criteria. This corresponds to the denominator of Equation 2. Next, all months in FY2021/22 and all institutions were flagged as to whether they used HIE. If at least one account within an institution used HIE for at least one day in a month, the institution was deemed to have used the HIE that month. Subsequently, for each facility type, we calculated the sum of the institutions that used HIE for each month. This corresponds to the numerator in Equation 2. Finally, for each facility type, we calculated the active institution ratio for each month using Equation 2.

Within facility types, hospitals were further classified based on the number of beds. Hospitals were divided into three categories: those with ≥200 beds, those with 100-199 beds, and those with ≤99 beds, and Equation 2 was calculated for each classification.

#### Monthly Active Institution Ratio of Medical Institutions and Man-Days of HIE Use for Each User Type

We compared the monthly active institution ratios at medical institutions for each HIE and the proportion of man-days of HIE use by user type. Equation 2 defines the monthly active institution ratio. “Medical institutions” include the facility types “hospital,” “medical clinic,” and “dental clinic.” We analyzed all HIEs during all periods for which data could be obtained.

The total number of medical institutions participating in each HIE on the last day of each month was calculated. Next, for each HIE in each month, each medical institution of each HIE was flagged as to whether it used the HIE. If at least one account from within the institution used the HIE for at least one day in a month, the institution was deemed to have used HIE that month. Subsequently, for each HIE in each month, we calculated the sum of the institutions that used it. We then calculated the active institution ratio of medical institutions for each HIE for each month, according to Equation 2.

Next, we classified all user occupation data into 8 user types: “doctor,” “nurse,” “rehabilitation staff,” “pharmacist,” “dental profession,” “nursing care staff,” “other medical professions,” and “type unknown.” We aggregated the total number of man-days of HIE use by user type and calculated the proportion of man-days by user type.

## Results

### Data Collection

The MHLW report listed 218 HIEs. However, numbers 57-60 in the report are federated, and the aggregated statistics are shown as number 61. After removing these duplicates, there were 214 HIEs listed [9]. Of the 214 HIEs, 36 HIEs were connected to more than 100 institutions and 45 were connected with more than 10,000 patients. Overall, 21 HIEs met both inclusion criteria. Considering number 61 to be 4 HIEs, 24 HIEs were considered candidates for data collection.

**Figure 1.**
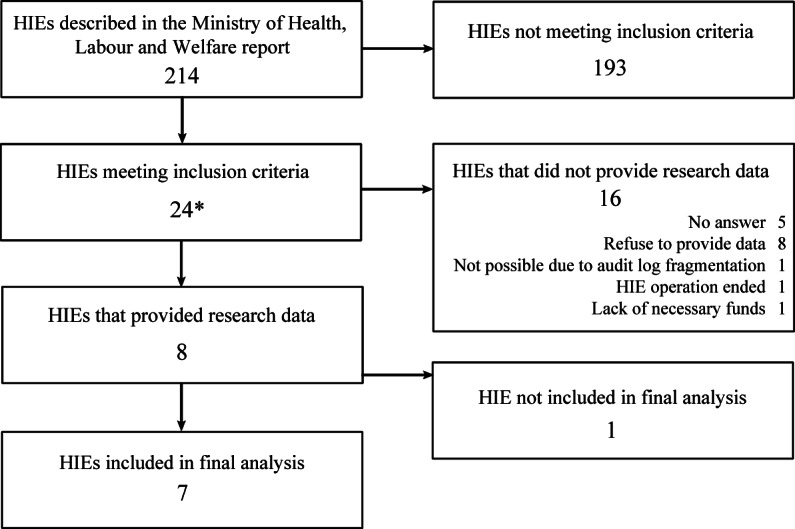
Flow diagram of HIE selection. HIE: health information exchange. *HIE number 61 was considered to be 4 HIEs.

Initially, we requested research cooperation from each RHIO administrator via email. The data request document is shown in Multimedia Appendix 2. Thereafter, we requested each RHIO to provide data through a web conference once we were able to have detailed discussions. We obtained research data from 8 HIEs. Although these tasks were sometimes performed free of charge, the extraction of access logs from 2 HIEs was performed for a fee. One of the 8 HIEs was unable to extract comprehensive audit log data of query-based exchange and was removed from the final analysis. The flow diagram is depicted in Figure 1. Among the 24 HIEs that met the inclusion criteria for this study, we calculated the average and standard deviation of the monthly active institution ratio based on the MHLW report separately for the HIEs that were included in the final analysis and those that were not. The results are shown in Table 1.

To avoid identification, all RHIOs and HIEs were assigned a pseudonym using letters from A to G. We obtained the audit log data from RHIO A, RHIO C, and RHIO E from April 2017 to March 2022. We obtained audit log data from April 2018 to March 2022 from RHIO B. From RHIO F and RHIO G, we obtained audit log data from April 2021 to March 2022. We obtained audit log data from October 2021 to March 2022 from RHIO D. HumanBridge [32] (developed by Fujitsu Limited) has an audit log extraction feature added by default. Therefore, audit log extraction in RHIOs that used HumanBridge was performed using this feature. The extraction of audit logs from HIEs other than HumanBridge was conducted by requesting the system vendor to extract audit logs from the RHIO secretariat.

**Table 1.** Average monthly active institution ratio based on the MHLW^a^ report.

	Health information exchange^b^, n	Monthly active institution ratio based on the MHLW report (%)^c^, mean (SD)
Included in the final analysis	7	46.1 (24.4)
Not included in the final analysis	17	46.4 (19.1)

a MHLW: Ministry of Health, Labour and Welfare.

b Among the health information exchanges listed in the MHLW report, those connected to more than 100 institutions and with more than 10,000 patients were included in this analysis.

c For each health information exchange, the MHLW report lists the number of participating medical institutions and the number of medical institutions that accessed health information exchange (ie, the number of institutions that used health information exchange during the month covered by the survey). We divided the number of medical institutions that accessed health information exchange by the number of participating medical institutions for each health information exchange and called this number “monthly active institution ratio based on the MHLW reports.”

Of the data items we attempted to obtain in advance (Textbox 1), the type of device used for access was not collected by any HIEs. Consequently, we could not analyze the type of device with which the HIE was accessed. We obtained users’ date of account registration and account deletion in the HIE from only 3 RHIOs. For the remaining 4 HIEs, we could not determine the exact number of registrants in each period.

### Characteristics of HIEs Included in the Final Analysis

All 7 HIEs began operations between 2010 and 2015. Of the 24 HIEs that met the inclusion criteria, 9 did not have membership fees, and of the 7 HIEs included in the final analysis, 3 did not have participation fees for connected institutions. Overall, 5 RHIOs adopted ID-Link for the HIEs they operate, 3 RHIOs adopted HumanBridge, and 2 RHIOs employ products other than these. The reason that the sum of these products is more than 7 is that some RHIOs operate multiple products in parallel.

For HIE A to G, the patient consent rates for HIE in 2022 were 21.2%, 5.6%, 69.5%, 1.9%, 2%, 4%, and 6.5%. Patient consent rate was obtained by dividing the number of patients connected with the HIE by the population of the area. All 6 HIEs except HIE C required patients to complete a paper consent form for their medical data to be viewed by health care workers using the HIE. HIE C uses paper consent forms as well as the “patient demographic data synchronization feature” provided by ID-Link. For institutions that disclose patient data to HIE C, basic patient profiles such as name, date of visit, and public insurance data are automatically accumulated in the HIE. This feature allows health care workers to use query-based exchange to obtain patient data in the event of emergency treatment, even if the patient cannot explicitly consent to the use of the HIE in advance. Patient enrollment in an HIE using this feature is opt-out, that is, individuals are considered to implicitly consent to participate in the HIE unless participation is explicitly declined. Therefore, the apparent consent rate of HIE C is extremely high.

### Percentage of Days of HIE Use by Each Hospital Doctor in FY2021/22

A total of 3 HIEs met the criteria: HIE A, HIE B, and HIE C. The number of hospital doctor accounts registered in HIEs operated by these 3 HIEs in FY2021/22 was 7833. For each of these doctor accounts, we calculated the percentage of days of HIE use according to Equation 1. The overall results are shown in Table 2.

**Table 2.** Percentage of days of HIE^a^ use by doctors affiliated with the hospital.

Days of HIE use, %	Hospital doctors, n
0	7326
>0 and ≤5	412
>5 and ≤10	39
>10 and ≤15	11
>15 and ≤20	17
>20 and ≤25	5
>25 and ≤30	4
>30 and ≤35	5
>35 and ≤40	5
>40 and ≤45	1
>45 and ≤50	1
>50	7

a HIE: health information exchange.

### Man-Days for Monthly HIE Use by Each Institution in FY2021/22

Different HIEs met the criteria for each facility type as shown in Table S2 in Multimedia Appendix 1. The cumulative number of months of hospital participation in HIEs in FY2021/22 was 3434. The distribution of man-days for monthly HIE use by hospitals is shown in Table 3. Table 4 shows the analysis results for facility types other than hospitals. The number of institutions connected to the HIEs included in the analysis for each month in FY2021/22 is shown in Table S3 in Multimedia Appendix 1.

**Table 3.** Distribution of man-days for monthly HIE^a^ use for hospitals.

Man-days for monthly HIE use	Cumulative number of months, n
0	1781
1-10	921
11-20	287
21‐30	129
31‐40	67
41‐50	59
51‐60	28
61‐70	14
71‐80	11
81‐90	21
91‐100	13
≥101	103

a HIE: health information exchange.

**Table 4.** Distribution of man-days for monthly HIE^a^ use by medical care institutions other than hospitals.

Man-days for monthly HIE use per institution	Months per institution, n (%)				
	Medical clinic (7791 months)	Dental clinic (1006 months)	Pharmacy (5140 months)	Visiting nursing station (983 months)	Nursing facility (3629 months)
0	5914 (75.9)	971 (96.5)	4081 (79.4)	672 (68.4)	2781 (76.6)
1‐5	1063 (13.6)	31 (3.1)	733 (14.3)	134 (13.6)	452 (12.5)
6‐10	261 (3.4)	1 (0.1)	116 (2.3)	42 (4.3)	126 (3.5)
11‐15	177 (2.3)	1 (0.1)	57 (1.1)	40 (4.1)	73 (2)
16‐20	137 (1.8)	2 (0.2)	80 (1.6)	19 (1.9)	48 (1.3)
21‐25	138 (1.8)	0 (0)	16 (0.3)	3 (0.3)	52 (1.4)
≥26	101 (1.3)	0 (0)	57 (1.1)	73 (7.4)	97 (2.7)

a HIE: health information exchange.

### Monthly Active Institution Ratio in FY2021/22

**Figure 2.**
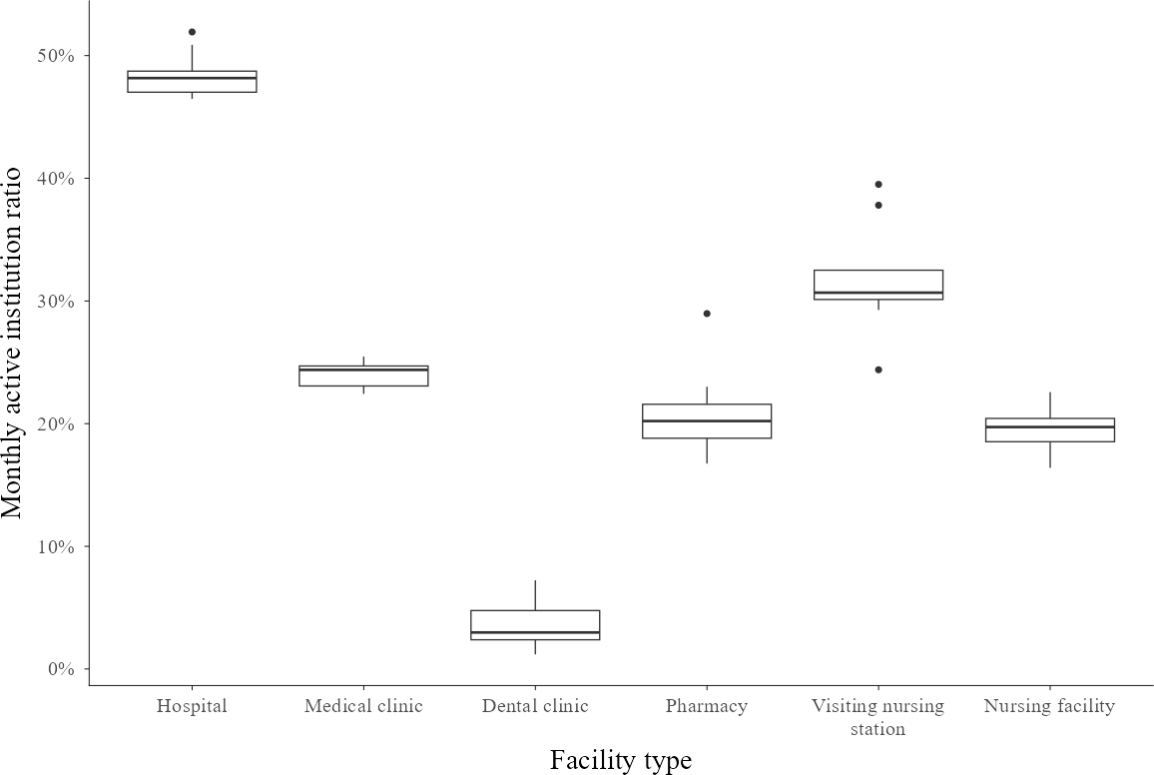
Monthly active institution ratio of health information exchange categorized by facility type.

Table S2 in Multimedia Appendix 1 lists the HIEs that met the criteria in this section. The median (IQR) monthly active institution ratios were 0.482 (0.470‐0.487) in hospitals, 0.244 (0.231‐0.247) in medical clinics, 0.030 (0.024‐0.048) in dental clinics, 0.202 (0.188‐0.216) in pharmacies, 0.307 (0.301‐0.325) at visiting nursing stations, and 0.197 (0.185‐0.204) in nursing facilities. We illustrated the monthly active institution ratios using box plots in Figure 2.

Table 5 shows the monthly active hospital rate for each category subdivided by the number of hospital beds. HIE F data could not be combined with hospital bed data and was therefore excluded from the analysis.

**Table 5.** Monthly active hospital ratio subcategorized by the number of hospital beds per institution.

Hospital beds, n	Monthly active institution ratio (%), median (IQR)
≤99	34.2 (30.9‐39.5)
100‐199	65.2 (65.2‐69.6)
≥200	75.0 (72.9‐77.1)

### Monthly Active Institution Ratio of Medical Institutions and Man-Days of HIE Use for Each User Type

We analyzed all 7 HIEs. As the period of audit log data obtained differs for each HIE, the analysis period also differs. A total of 5 HIEs included all types of medical institutions; however, no dental clinics participated in HIE D and HIE E. Regarding user type data, precise data were not available for HIE D. Occupational data for HIE F could only be obtained for “doctors” and “other medical professions.”

Some HIEs have restrictions on the types of users that can use the HIE. In 2 HIEs, users affiliated with medical clinics could only use the HIEs if they were doctors. In one HIE, users who were affiliated with hospitals that did not disclose patient data to the HIE could only use the HIE if they were doctors. In addition, 6 HIEs had no restrictions on the type of data that authorized health care workers could view. One HIE was set up so that people other than doctors affiliated with the hospital could not view outpatient treatment data.

**Figure 3.**
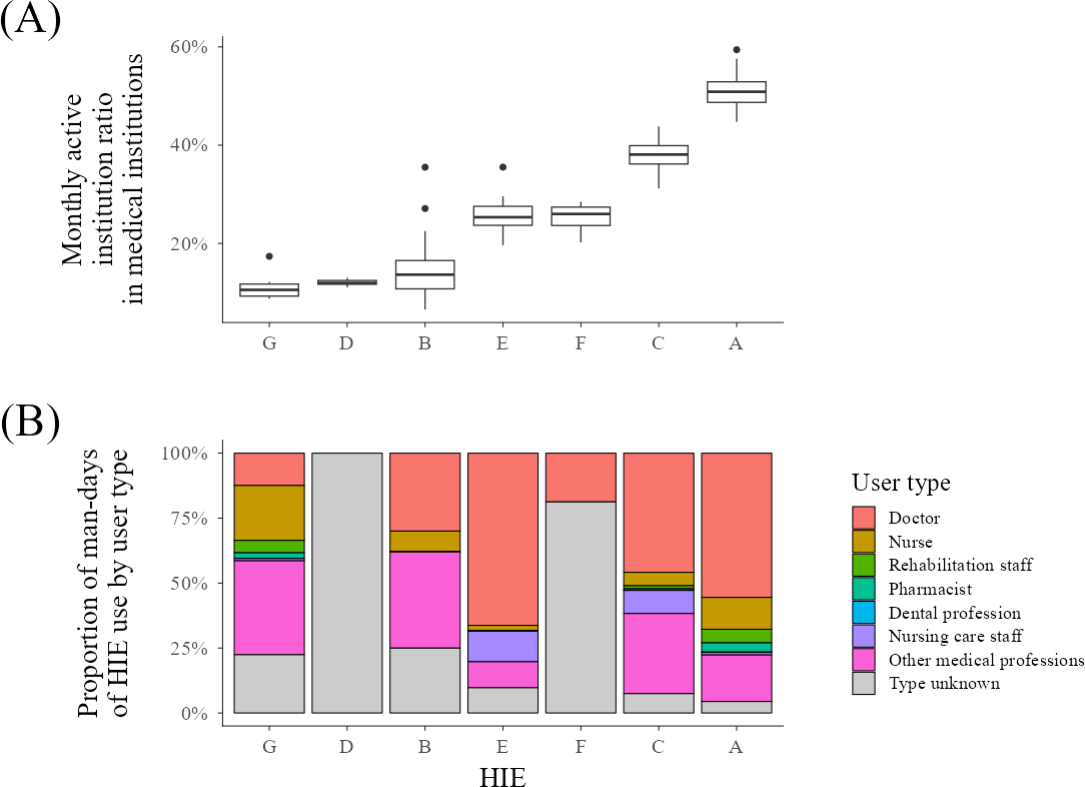
(A) Monthly active institution ratio and (B) proportion of man-days of HIE use by user type for each HIE. HIE: health information exchange.

We illustrated the monthly active institution ratio of HIE and the proportion of man-days of HIE use by user type in medical institutions using box plots and bar graphs (Figure 3).

Monthly active institution ratios of HIE in medical institutions are also shown in Table S4 in Multimedia Appendix 1. The proportions of man-days of HIE use by user type in medical institutions are also shown in Table S5 in Multimedia Appendix 1.

## Discussion

### Overview of HIE Use

As already mentioned, the low utilization of large-scale HIEs has previously been suggested by MHLW reports (Table S1 in Multimedia Appendix 1). Our analysis included data from only 18% (7/32) of such HIEs. However, among HIEs that met the inclusion criteria, monthly active institution ratios based on the MHLW report were similar for HIEs included in the final analysis and those not included (Table 1). This suggests that the results of this study apply to some extent to large-scale HIEs in general. However, HIEs with fewer participating institutions have a higher monthly active institution ratio based on the MHLW report than large-scale HIEs (Table S1 in Multimedia Appendix 1). Therefore, it is possible that small-scale HIEs are used more actively than the results of this study suggest. However, the MHLW report includes 13 HIEs where the number of participating medical institutions is 1 or 2, and the number of participating institutions is equal to the number of institutions that have accessed HIEs. The active institution ratio based on the MHLW report should be interpreted with caution because extremely small-scale HIEs are driving up the ratio.

Across the large-scale HIEs analyzed in this study, many health care workers and institutions did not use query-based exchange. These results are consistent with MHLW reports (Table S1 in Multimedia Appendix 1). We found that 93.5% (7326/7833) of the doctors at hospitals registered with HIEs do not use them even once per year (Table 2). In addition, 51.9% (1781/3434) of hospitals did not use query-based exchange even once per month (Table 3). This is lower than the values reported by previous studies conducted in other countries [20,33]. The monthly active institution ratio is higher for hospitals with more beds. The median (IQR) monthly active institution ratio for hospitals with ≤99 beds is 34.2% (30.9%-39.5%), but it is 75% (72.9%-77.1%) for hospitals with ≥200 beds (Table 5). Among facilities other than hospitals, the monthly active institution ratio is even lower than for hospitals with ≤99 beds. The median (IQR) monthly active institution ratio for visiting nursing stations reached 30.7% (30.1%‐32.5%), but it was only approximately 20% for medical clinics, pharmacies, and nursing care facilities (Figure 2). As for dental clinics, the median (IQR) monthly active institution ratio was only 3% (2.4%-4.8%). Previous studies outside Japan have also revealed that HIEs are not often used by dental practices [34]. This is the first study to reveal the active institution ratio of HIE by facility type in Japan.

Where query-based exchange was used, most people and institutions only used it for a limited number of days. Previous reports have not provided user-level analysis; therefore, this study is the first to reveal the total number of days of HIE use by health care workers and institutions. Of the 507 hospital-affiliated doctor accounts that actively used query-based exchange in FY2021/22, we found that 81.3% (412/507) used it for 5% or fewer days (Table 2). In other words, assuming the average doctor works 20 days per month, most of these doctors use query-based exchange less than once per month. As the percentage of days of HIE use increases, the number of corresponding hospital doctor accounts tends to decrease. This trend is reflected in man-days for monthly HIE use of hospitals. We found that 90.8% (3118/3434) of all hospitals use HIE for 30 or fewer man-days per month (Table 3). In these hospitals, query-based exchange is used by less than one user daily. The number of man-days of HIE use in hospitals also shows a tendency for the number of applicable months to decrease as the number of man-days increases. This trend remains true for man-days for monthly HIE use at facilities other than hospitals (Table 4). However, some institutions and users use query-based exchange for many days. Of the hospital-affiliated physician accounts, 19 users used query-based exchange for 30% or more days. Of the cumulative months of hospital participation in HIEs in FY2021/22, we found that 3% (103/3434) had over 101 man-days for HIE use (Table 3). This exceeds the number of months when man-days of monthly HIE use are in the range of 91-100. For visiting nursing stations and nursing care facilities, the number of months in which the number of man-days for HIE use exceeds 26 is greater than the number of months in which the number of man-days is 21-25 (Table 4), showing there are significant disparities in HIE use across institutions and users.

Monthly active institution ratios for medical institutions vary widely by HIE. HIE A, which has the highest monthly active institution ratio, has a median rate of 50.9% (IQR 48.7%-52.9%), but some HIEs have a rate of over 10% (Figure 3, Table S4 in Multimedia Appendix 1). The proportion of man-days of HIE use by each user type is not constant for each HIE. However, regarding the HIEs that could be confirmed, the number of man-days of HIE use was low for dental professionals, pharmacists, and rehabilitation workers.

### Possible Factors Influencing HIE Use

Many other factors could have influenced monthly active institution ratios and man-days of monthly HIE use, for example, the system used and whether there are membership fees and usage restrictions based on use type. The data viewed by health care professionals when using HIEs is also extremely important. None of these can be shown as individual HIE data due to privacy considerations; therefore, they cannot be discussed in relation to the results shown in Figure 3. This is a significant limitation of this study and indicates the need for further research into why there are such large disparities in demand for HIE in Japan.

Although a detailed elucidation must be reserved for future research, two factors may have influenced the monthly active institution ratio of facilities in the HIEs in our analysis. One is the consent rate of patients to participate in HIEs. HIE A, which had the highest monthly active institution ratio among medical institutions in this analysis, had a relatively high patient consent rate of over 21%. HIE C, which had the second highest monthly active institution ratio, has a partial opt-out policy and a very high consent rate. However, HIEs G, D, and B, where the monthly active institution ratio was 20% or less, had patient consent rates of 7% or less. The patient consent rate in HIEs E and F, where the monthly active institution ratio was in the 20% range, was less than 5%, and therefore lower than in G and B. High consent rates may have contributed to the high active institution ratios for HIEs A and C, but this study cannot determine whether these factors are causally related.

Another factor that may have influenced the monthly active institution ratio is the number of staff at each participating institution. As already shown, the active institution ratio was higher in hospitals with ≤99 beds than in medical clinics, and it was higher in hospitals with ≥200 beds than in hospitals with ≤99 beds. It is natural to assume that this difference is caused by the absolute number of staff working at each institution. Therefore, when considering the active institution ratio for a given HIE, the value is likely to be high if a large proportion of the institutions participating in the HIE are large hospitals.

### Audit Log for Further Research Analysis

To analyze HIE use in detail, proper design of the audit log is extremely important. When analyzing the audit logs in this study, two characteristics of some audit logs posed obstacles. One was that the extracted audit logs are not comprehensive. In HIEs that were configured using products from multiple vendors [35], each product generally had its own unique audit log design and storage. To perform detailed analysis of the usage of such HIEs, it was necessary to extract the logs from each product. This was difficult when each product was controlled by a different institution; specifically, the HIE platform system is managed by the RHIO, but the EMR data viewing system may be managed by each hospital. In this case, we need to obtain consent for research collaboration from each institution to perform overall log extraction. If user access to individual systems via the platform system is recorded in the audit log, analyzing the general usage status may be possible by extracting only the platform system’s audit log. However, in practice, access to individual systems is not necessarily recorded in the platform system’s audit log. In HIEs configured using products from multiple vendors, careful attention must be paid to facilitating comprehensive log extraction.

The second obstacle was the incompatibility of the institution IDs and user IDs used in HIEs. To clarify the factors that create the disparities in HIE usage across users and across institutions, more detailed data on users and medical institutions were required, such as medical specialty and whether institutions provide acute or chronic care. However, such data are not generally included in the audit log itself; therefore, log data needs to be cross-referenced with institution IDs compiled in the master dataset by the government or the detailed user data of each institution. As the institution IDs used in the audit log data extracted in this study did not necessarily correspond to the institution IDs assigned by the MHLW, it was difficult to cross-reference log data with other datasets. To investigate HIE usage in greater depth than this study, it is recommended to use master data that can be matched with EMR and official datasets when designing audit logs.

### Limitations

This study had several limitations. The most significant limitation was the need to maintain the anonymity of the HIEs included in the analysis. Therefore, important data, such as the systems employed by individual HIEs and the types of medical data disclosed, were either kept private or disclosed anonymously. Consequently, it was almost impossible to analyze the causes of differences in active institution ratios for individual HIEs from the data. We also attempted to evaluate the viewing situation for all data types, such as images and prescriptions. However, it was difficult to perform a comprehensive analysis because the data storage format was not standardized for each HIE. The list of HIEs included in the analysis differed for each analysis because the data items that could be obtained differed for each HIE. Data regarding the type of device used to access the HIE could not be obtained from any HIE.

Some HIEs have features other than viewing patient medical data, such as sending and receiving documents or messages [8]. Previous reports indicated that some HIEs actively used these additional features when treating COVID-19 patients [27]. As this study focused on query-based exchange, we did not perform a quantitative analysis of the usage of other features. Another study is required on the actual usage of features other than query-based exchange.

This study revealed that most users do not use query-based exchange or use it infrequently, but it is impossible to prove whether this is due to a lack of patients’ medical data in the HIE repository or a lack of need to view data. As mentioned above, HIEs vary widely in both patient consent rates and the types of data that health care professionals can view. Therefore, it is difficult to provide a single answer to this remaining question. To answer this question, a deeper investigation of each HIE is required, using more detailed audit log analysis, system descriptions, and qualitative research.

### Conclusions

In the large-scale HIEs surveyed in this study, the overall usage of the on-demand patient data viewing feature was low, consistent with past MHLW reports. User-level analysis of audit logs revealed large disparities in the number of days of HIE use among health care workers and institutions. There were also large disparities in HIE use by facility type or HIE, and the percentage of cumulative HIE usage days by user type also differed by HIE. This study indicates the need for further research into why there are large disparities in demand for HIEs in Japan, as well as the need to design comprehensive audit logs that can be matched with other official datasets.

## Supplementary material

10.2196/56263 Multimedia Appendix 1 Detailed tables of data analysis results.

10.2196/56263 Multimedia Appendix 2 Request letters to health information exchange operators [document in Japanese].

## Abbreviations

EMR: electronic medical record
FY: financial year
HIE: health information exchange
MHLW: Ministry of Health, Labour and Welfare
RHIO: regional health information organization
RHN: regional health care network
