# Supplementary material for: Health Care Worker Usage of Large-Scale Health Information Exchanges in Japan: User-Level Audit Log Analysis Study
Source: JMIR Med Inform. 2024 Oct 9;12:e56263. doi: 10.2196/56263 (PMC11481819; doi:10.2196/56263)
Supplement: Multimedia Appendix 1 [file medinform-v12-e56263-s001.docx]

# Multimedia Appendix 1

Table S1. Monthly active institution ratio based on the MHLW report. HIEs were classified by the number of connected institutions.

| Characteristic of HIEs | | Number of HIEs | Monthly active institution ratio based on the MHLW report (%)^a^ |
| --- | --- | --- | --- |
|  |  | N | Mean (SD) |
|  |  |  |  |
| **Connected institutions (N)^b^** |  |  |  |
|  | ≤9 | 64 | 75.9 (27.2) |
|  | 10–99 | 115 | 50.2 (30.2) |
|  | ≥100 | 32 | 38.1 (19.9) |

^a^The MHLW report lists the “number of participating medical institutions” and the “number of medical institutions that accessed HIE” for each HIE. According to the MHLW report, the “number of medical institutions that accessed HIE” is the number of institutions that used HIE during the month covered by the survey. Referring to the report, we divided “the number of medical institutions that accessed HIE” by “the number of participating medical institutions” in each HIE and named this number "monthly active institution ratio based on the MHLW reports."

^b^We classified HIEs into three groups based on the number of connected institutions. The number of connected institutions is based on the "number of participating medical institutions" in the MHLW report. Of the 218 HIEs listed in the MHLW report, 1 federated HIE (No. 61), 5 HIEs for which the actual number of accessed institutions was unknown (No. 115, 124, 169, 200, 217), and 1 remote conference system (No. 121) were excluded from the analysis.

Table S2. HIEs included in each analysis.

| Facility type to be analyzed | Analysis | |
| --- | --- | --- |
|  | Man-day of monthly HIE use by each institution in FY2021/22 | Monthly active institution ratio in FY2021/22 |
|  | Pseudonym of HIE | Pseudonym of HIE |
|  |  |  |
| Hospital | A B C E F G | A B C E F G |
| Medical Clinic | A B C E F | A B C E F |
| Dental Clinic | A B C E F | A B C E F |
| Pharmacy | A B C F G | A B C F G |
| Visiting Nursing Station | A C F | A C F |
| Nursing Facility | A C F | A C F |

Table S3. Number of institutions connected to the HIEs included in the analysis in each month of FY2021/22.

| Year and month | Number of connected institutions | | | | | |
| --- | --- | --- | --- | --- | --- | --- |
|  | Hospital | Medical clinic | Dental clinic | Pharmacy | Visiting nursing station | Nursing facility |
| Month, year | N^a^ | N^a^ | N^a^ | N^a^ | N^a^ | N^a^ |
|  |  |  |  |  |  |  |
| April, 2021 | 285 | 647 | 83 | 426 | 81 | 294 |
| May, 2021 | 285 | 647 | 83 | 427 | 82 | 296 |
| June, 2021 | 285 | 651 | 84 | 428 | 82 | 296 |
| July, 2021 | 285 | 651 | 84 | 428 | 83 | 297 |
| August, 2021 | 285 | 646 | 84 | 429 | 83 | 298 |
| September, 2021 | 284 | 647 | 84 | 428 | 83 | 298 |
| October, 2021 | 285 | 648 | 84 | 427 | 82 | 299 |
| November, 2021 | 286 | 648 | 84 | 428 | 83 | 302 |
| December,2021 | 288 | 648 | 84 | 429 | 82 | 306 |
| January, 2022 | 288 | 651 | 84 | 430 | 81 | 310 |
| February, 2022 | 289 | 654 | 84 | 430 | 81 | 312 |
| March, 2022 | 289 | 653 | 84 | 430 | 80 | 321 |

^a^The number of connected institutions as of the last day of each month.

Table S4. Monthly active institution ratio of medical institutions by HIEs.

| HIE | G, N = 12^1^ | D, N = 6^1^ | B, N = 48^1^ | E, N = 60^1^ | F, N = 12^1^ | C, N = 60^1^ | A, N = 60^1^ |
| --- | --- | --- | --- | --- | --- | --- | --- |
|  |  |  |  |  |  |  |  |
| Usage Rate | 0.105 (0.093, 0.117) | 0.119 (0.117, 0.125) | 0.136 (0.108, 0.165) | 0.253 (0.237, 0.276) | 0.260 (0.236, 0.274) | 0.381 (0.362, 0.399) | 0.509 (0.487, 0.529) |
| ^1^Median (IQR) | | | | | | | |

Table S5. The proportion of man-days of HIE use by user type.

| User type | Rate of man-days | | | | | | |
| --- | --- | --- | --- | --- | --- | --- | --- |
|  | HIE G | HIE D | HIE B | HIE E | HIE F | HIE C | HIE A |
|  | % | % | % | % | % | % | % |
|  |  |  |  |  |  |  |  |
| Doctor | 12.4 | - | 30.0 | 66.3 | 18.8 | 45.9 | 55.5 |
| Nurse | 21.1 | - | 7.9 | 2.0 | - | 5.1 | 12.3 |
| Rehabilitation job | 4.8 | - | 0.1 | - | - | 1.2 | 5.2 |
| Pharmacist | 2.1 | - | - | 0.3 | - | 0.6 | 3.5 |
| Dental profession | 0.1 | - | - | - | - | 0.1 | 0.2 |
| Nursing care occupation | 0.9 | - | 0.1 | 11.7 | - | 8.9 | 1.0 |
| Other medical occupations | 36.2 | - | 37.0 | 10.0 | - | 30.9 | 17.9 |
| Occupation unknown | 22.4 | 100 | 25.0 | 9.7 | 81.2 | 7.5 | 4.4 |
