# Supplementary material for: Health Care Worker Usage of Large-Scale Health Information Exchanges in Japan: User-Level Audit Log Analysis Study
Source: JMIR Med Inform. 2024 Oct 9;12:e56263. doi: 10.2196/56263 (PMC11481819; doi:10.2196/56263)
Supplement: Multimedia Appendix 2 [file medinform-v12-e56263-s002.pdf]

[地域医療情報連携ネットワーク名］事務局御中

京都大学 黒田知宏 と申します。

当方では、EHR（HIE）の普及要因（普及阻害要因）を探る研究を
計画しております。

先日本院の倫理委員会にて研究計画書が承認されたことをうけて、
登録患者数 1 万人以上、かつ参加医療機関数 100 以上の地域ネット
に研究の協力を御願いしているところです。
ついては、貴ネットワークにも、調査にご協力頂ければと思い
ご連絡を差し上げました。

お忙しいところお手数をおかけして申し訳ありませんが、
お力添えを賜りたく、よろしくお願いいたします。

具体的な研究内容については、オンライン会議等でお伝えできればと存じますので、
ご連絡させて頂いてよい方などをご紹介頂きたく、よろしくお願い申し上げます。

===================
黒田知宏
　京都大学 教授
　　医学部附属病院 医療情報企画部長・病院長補佐
　　医学研究科　医学・医科学専攻 医療情報学分野
　　情報学研究科　社会情報学専攻 医療情報学講座
===================
Tomohiro Kuroda
Professor
 Graduate School of Medicine
 Graduate School of Informatics
Director / CIO
 Div. Medical IT & Admin. Plan.
Kyoto University Hospital
